# Klotho Contributes to Pravastatin Effect on Suppressing IL-6 Production in Endothelial Cells

**DOI:** 10.1155/2016/2193210

**Published:** 2016-02-29

**Authors:** Weiwei Xia, Aihua Zhang, Zhanjun Jia, Jun Gu, Hongbing Chen

**Affiliations:** ^1^Department of Clinical Laboratory, Nanjing Children's Hospital, Nanjing Medical University, Nanjing 210008, China; ^2^Jiangsu Key Laboratory of Pediatrics, Nanjing 210029, China; ^3^State Key Laboratory of Protein and Plant Gene Research, College of Life Science, Peking University, Beijing 100871, China

## Abstract

Both statins and klotho have been shown to be beneficial in vascular diseases. Interleukin- (IL-) 6 is evidenced as an indicator reflecting the stability of atherosclerotic plaque and involved in the pathogenesis of artery atherosclerosis. However, the relationship between statin, klotho, and IL-6 under an inflammatory environment is unknown. Using primary human umbilical vein endothelial cells (HUVECs), pravastatin dose-dependently induced klotho expression in contrast to remarkable suppression to IL-6 expressions determined by qRT-PCR. Moreover, TNF-*α*-induced IL-6 was partly but significantly blunted by pravastatin detected by ELISA. To further study the role of klotho in modulating IL-6 expression, endothelial cells with klotho overexpression were treated with TNF-*α*. Importantly, TNF-*α*-induced IL-6 production was markedly attenuated in klotho-overexpressed cells. In agreement with in vitro data, a marked reduction of klotho mRNA expression was found in isolated peripheral blood mononuclear cells (PBMCs) from patients with atherosclerosis. Together, these data suggested that pravastatin could suppress IL-6 production via promoting klotho expression in endothelial cells under inflammatory stimuli.

## 1. Introduction

The chief cause of cardiovascular disease is the formation of atherosclerotic plaque possibly due to the sustained elevation of blood cholesterol and subsequent inflammatory response in vascular region [1, 2]. Statins such as pravastatin, lovastatin, and simvastatin are widely used in the clinic for the lowering of blood cholesterol to reduce the risk of cardiovascular diseases [3, 4]. Recently, the anti-inflammatory effect of statins was defined as a protective property of this kind of drugs in benefiting cardiovascular system independently of the action in modulating lipid metabolism [5, 6].

Klotho has been identified as a kidney-generated product and is of vital importance in regulating calcium-phosphorus metabolism, aging, organ injuries, and so on [7, 8]. Klotho was also reported to have a role in modulating inflammation [9]. For example, klotho reduction may promote inflammation in chronic obstructive pulmonary disease and aging-related kidney injury [10, 11]. More interestingly, recent evidence demonstrated that statins could enhance klotho expression in renal cells [12]. However, whether statins regulate klotho in vascular cells is still undefined.

IL-6 is a proinflammatory cytokine with multiple functions and was reported to serve as an indicator to reflect the stability of atherosclerotic plaques [13, 14]. Moreover, evidence also highly suggested that IL-6 contributes to the pathogenesis of artery atherosclerosis via promoting local inflammatory lesions [13]. Recently, a novel therapeutic reagent targeting IL-6 signaling is under the clinical trial for the treatment of chronic inflammatory diseases including atherosclerosis [15]. In the current study, we investigated pravastatin effect on regulating klotho and Il-6, as well as the relationships between them. The findings not only increased our understanding on the protective property of statins in cardiovascular system but also suggested some potential in treating atherosclerosis by targeting klotho.

## 2. Methods

### 2.1. Reagents and Plasmid

Pravastatin and TNF-*α* were purchased from Sigma (Catalog #: T0157). Human IL-6 ELISA kit was from Boster (Catalog #: EK0411). Goat anti-klotho (T-19) polyclonal was from Santa Cruz Biotechnology (Catalog #: sc-22218). Apoptosis detection kit was from BD Biosciences (Catalog #: BD556547). The full-length complementary DNA of the human secreted form of klotho was amplified using specific primers and total cDNA from human kidney. Then, the PCR product was subcloned into a FLAG-tag vector modified from pcDNA3 vector (Invitrogen). The pcDNA3 vector served as a backbone vector. The human klotho gene ID is 9365 and the length of the secreted form of klotho is 549 AAs (amino acids 1–549).

### 2.2. Primary Culture of HUVEC and Klotho Transfection

Human umbilical cord vein endothelial cells were isolated as described by Jaffe et al. [16]. Human umbilical cord veins were incubated with 0.2% collagenase for 15 min at 37°C. The solution containing the endothelial cells was collected from the cord by flushing with 30 mL buffer in a 50 mL sterile centrifuge tube [16] The cells were collected and cultured in medium 199 with 20% fetal bovine serum (FBS). For the klotho transfection, we used jet PEI*™*-HUVEC (Polyplus-transfection, # 133139) according to manufacturer's instruction. After 24-hour incubation with TNF-*α* or vehicle, cells and supernatants were harvested for Western blotting and ELISA analyses.

### 2.3. Isolation of Human PBMCs

Human blood (5 mL) was collected in tubes with heparin. Then, the blood was diluted with 5 mL Hanks's buffer and transferred into a new tube containing 10 mL human Lymphocytes separation medium (TBD). After centrifuging at room temperature for 15 min at 2000 rpm, PBMC layer was transferred into one new tube. Washing for three times with PBS, cells were resuspended, counted, and cultured with RPMI-1640 with 10% FBS.

### 2.4. Regular and Quantitative Real-Time PCR (qRT-PCR)

Total RNA from HUVEC and PBMC was extracted by TRIzol reagent (Invitrogen, Carlsbad, CA). Oligonucleotides were designed using the Primer3 software (available at http://frodo.wi.mit.edu/) and synthesized by Invitrogen company. The primer sequences are as follows: human IL-6: 5′-TGACCCAACCACAAATGC-3′ 3′-CTGGCTCTGAAACAAAGGAT-5′; human klotho: 5′-TCAGGCAAGATAAACCAA-3′ 3′-TCTAACAAACGGGAACG-5′; human *β*-actin: 5′-GTGGACATCCGCAAAGAC-3′ 3′-AAAGGGTGTAACGCAACTAA-5′. qRT-PCR was used to detect target gene expression. Reverse transcription was performed by using Transcriptor First-Strand cDNA Synthesis kit (Roche, Germany) according to the manufacture's instruction. Real-time PCR amplification was performed using the ABI 7500 real-time PCR Detection System (Foster City, CA) with FastStart Universal SYBR Green Master mix (Roche, Germany). Cycling conditions were 95°C for 10 min followed by 40 repeats of 95°C for 15 s and 60°C for 1 min. The relative gene expression level was calculated through delta-delta Ct method and *β*-actin was used as the internal control. Experiments were repeated in triplicate.

### 2.5. Western Blotting

Cells were washed with PBS and lysed in lysis buffer (20 mM Tris, pH 7.5, 150 mM NaCl, 1% triton X-100, 1 mM EDTA, 10 *μ*g/mL aprotinin, 10 *μ*g/mL leupeptin, and 1 mM phenylmethylsulfonyl fluoride). SDS-PAGE gel was used to separate protein. Next, the gel was transferred to PVDF membrane which was blocked with 5% nonfat milk in TBST. Immunoblotting was performed with primary antibody against klotho (1 : 1000). Blots were probed with primary antibody and followed with HRP-conjugated secondary antibody. The signal was detected by using chemiluminescent ECL reagent kit (Millipore).

### 2.6. ELISA Assay

The supernatants of HUVECs were collected after treatment as indicated. IL-6 level was measured by ELISA kit (Boster) following the manufacturer's instruction.

### 2.7. Apoptosis Assay

HUVEC cells were stained with FITC-annexin V and propidium iodide according to the instruction of manufacturer. Stained cells were analyzed using a BD FACSCalibur flow cytometer (Bedford, MA) and data analysis was performed with FlowJo software.

### 2.8. Statistical Analysis

All data are shown as means ± SDs. Analysis of variance (ANOVA) was followed by Newman-Keuls test to determine the statistical significance of differences between mean values. The computer program GraphPad Prism (GraphPad Software Inc., San Diego, CA, USA) was used for statistical analysis. Differences were considered significant at *P* < 0.05.

## 3. Results

### 3.1. Pravastatin Enhanced Klotho Expression in HUVECs

**Figure 1:**
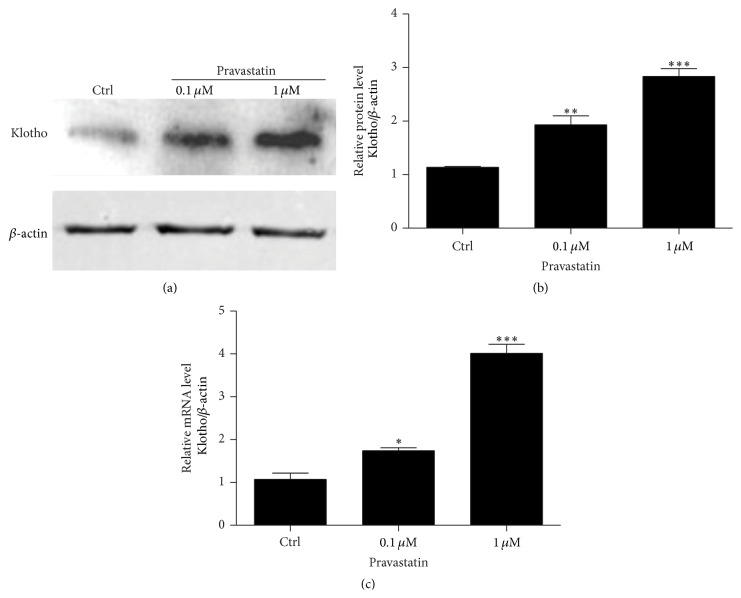
Pravastatin induced klotho expression in HUVECs. (a) Western blotting analysis of klotho in HUVECs. (b) Densitometric analysis of klotho Western blots. (c) Using qRT-PCR, pravastatin effect on klotho mRNA expression in HUVECs was determined. The values represent the means ± SDs (*n* = 4 in each group). ^*∗*^ *p* < 0.05 versus Ctrl group. ^*∗∗∗*^ *p* < 0.001 versus Ctrl group.

In order to test the effect of pravastatin on klotho regulation in HUVECs, we observed the protein and mRNA expressions of klotho following pravastatin administration. As shown by the data, pravastatin at a lower dose of 0.1 *μ*M resulted in a moderate but significant elevation of klotho protein by 2-fold (Figures 1(a) and 1(b)). More strikingly, a higher dose of pravastatin (1 *μ*M) led to a much greater induction of klotho by more than 2.8-fold (Figures 1(a) and 1(b)). Meantime, the mRNA regulation of klotho followed a similar pattern as its protein expression (Figure 1(c)). This finding not only demonstrated a direct role of pravastatin in upregulating klotho in vascular endothelial cells, but also suggested that klotho may contribute to the beneficial effect of pravastatin on vascular diseases to some extent.

### 3.2. Pravastatin Reduced IL-6 Expression in HUVECs

**Figure 2:**
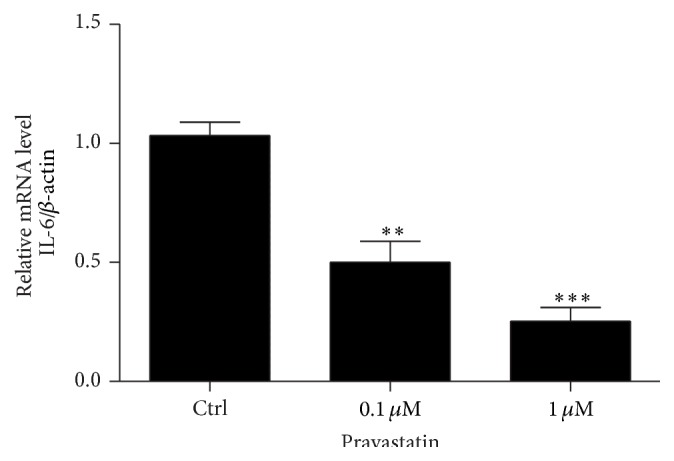
Pravastatin reduced IL-6 mRNA expression in HUVECs. The values represent the means ± SDs (*n* = 4 in each group). ^*∗∗*^ *p* < 0.01 versus Ctrl group. ^*∗∗∗*^ *p* < 0.001 versus Ctrl group.

Pravastatin was subjected to the primary HUVECs. After 24 hours, mRNA expression of IL-6 was examined using qRT-PCR. As shown in Figure 2. pravastatin at a dose of 0.1 *μ*M significantly reduced IL-6 mRNA expression by 45% and a higher dose of 1 *μ*M further downregulated IL-6 by 75%. The striking results indicated a potent activity of pravastatin in suppressing IL-6 production in HUVECs.

### 3.3. Pravastatin Attenuated TNF-*α*-Induced IL-6 Production in HUVECs

**Figure 3:**
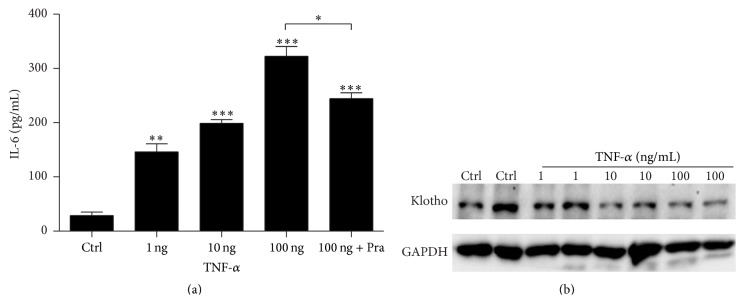
Pravastatin suppressed IL-6 production in HUVECs following TNF-*α* administration. (a) ELLISA assay of IL-6 in medium. (b) Western blotting analysis of klotho in HUVECs following TNF-*α* treatment. The values represent the means ± SDs (*n* = 4 in each group). ^*∗*^ *p* < 0.05 versus TNF-*α* 100 ng group. ^*∗∗*^ *p* < 0.01 versus Ctrl group. ^*∗∗∗*^ *p* < 0.001 versus Ctrl group.

To further examine pravastatin effect on inhibiting IL-6 production in activated HUVECs, an inflammatory cytokine TNF-*α* was used to treat HUVECs. As expected, TNF-*α* dose-dependently (0 ng, 1 ng, 10 ng, and 100 ng) increased IL-6 production (Figure 3(a)). In contrast to the elevation of IL-6, klotho was dose-dependently reduced by TNF-*α* (Figure 3(b)). Importantly, after application of pravastatin to HUVECs treated with 100 ng TNF-*α*, IL-6 production was significantly blunted (Figure 3(a)), indicating the efficacy of pravastatin in suppressing IL-6 production in HUVECs under inflammatory condition.

### 3.4. Overexpression of Klotho Blunted TNF-*α*-Induced IL-6 Production

**Figure 4:**
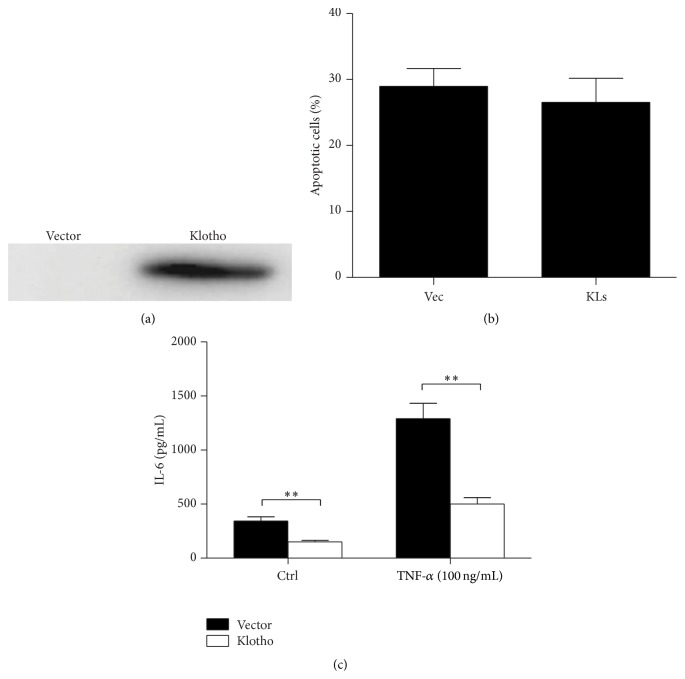
Overexpression of klotho in HUVECs reduced IL-6 production with or without TNF-*α* administration. (a) Western blotting analysis demonstrated a robust overexpression of klotho in HUVECs. (b) Apoptotic analysis. (c) ELLISA assay of IL-6 in medium of HUVECs with or without TNF-*α* treatment. The values represent the means ± SDs (*n* = 4 in each group). ^*∗∗*^ *p* < 0.01 versus vector group.

In consideration of the known evidence showing an anti-inflammatory property of klotho [9, 17], we overexpressed klotho gene by transfecting klotho plasmids into HUVECs, which did not affect cell apoptosis (Figures 4(a) and 4(b)). Strikingly, overexpression of klotho in cells without TNF-*α* administration significantly reduced IL-6 production by 52% (Figure 4(c)). Following the treatment of TNF-*α*, IL-6 secretion was elevated by more than 3-fold (Figure 4(c)). More importantly, such an induction of IL-6 was remarkably blunted by 60% in cells with klotho overexpression (Figure 4(c)). This data provided direct evidence showing that klotho could suppress IL-6 production in cells with or without inflammatory challenge.

### 3.5. Klotho mRNA Was Decreased in PBMCs from Atherosclerotic Patients

**Figure 5:**
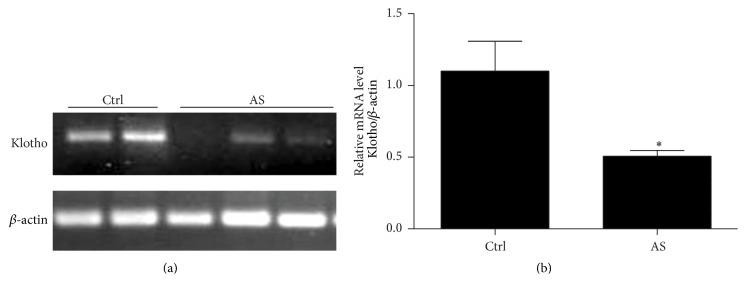
Decreased klotho mRNA expression in PBMCs from atherosclerotic patients. (a) PCR analysis of klotho mRNA expression. (b) Densitometric analysis of klotho PCR results. The values represent the means ± SDs (*n* = 4 in each group). ^*∗*^ *p* < 0.05 versus Ctrl group.

To investigate the involvement of klotho reduction in atherosclerosis, we isolated PBMCs from patients with artery atherosclerosis and examined the mRNA level of klotho using a regular PCR. As shown in Figures 5(a) and 5(b), PBMCs from atherosclerotic patients displayed a lower klotho mRNA expression compared with cells from healthy controls. This data indicated that the reduced klotho level in atherosclerotic patients might play a role in promoting IL-6 production and inflammatory response in both circulation and local atherosclerotic regions.

## 4. Discussion

Atherosclerosis featured by chronic inflammation is a common cause of cardiovascular diseases [2, 18]. During the formation of atherosclerosis in vasculature, hyperlipidemia was thought as the key insult triggering the inflammatory lesions [19]. In the past years, statins, a class of potent drugs in lowering blood cholesterol, were widely used in clinic for the treatment of hyperlipidemia and the prevention of cardiovascular diseases [3, 4]. With the rapid progress of clinical and basic research, statins were found to be anti-inflammatory independently of their fundamental properties in modulating lipid metabolism [5, 6]. Among a number of proinflammatory cytokines, IL-6 was not only an important contributor of the pathogenesis of atherosclerosis, but also a valuable indicator of the atherogenesis and instability of atherosclerotic plaques [13, 14]. Clinical studies convincingly demonstrated the elevation of IL-6 in patients with atherosclerosis [20]. In the present study, we found a remarkable capability of pravastatin in suppressing IL-6 in endothelial cells with or without stimulation of TNF-*α*. Endothelial cell serves as a major target of inflammatory insults. Inflammation resulted from both inflammatory cells and damaged vascular cells contribute to the vascular lesions and the formation of atherosclerotic plaques [21, 22]. The downregulation of IL-6 by pravastatin could be beneficial in antagonizing the progression of atherosclerotic lesions and plaque instability.

Klotho is chiefly produced by kidney and exerts multifunctions in calcium-phosphorus metabolism, aging, organ injuries, and so on [7, 8]. Emerging evidence demonstrated an anti-inflammatory action of klotho under pathological conditions [9, 23]. In this study, we observed a significant reduction of klotho in PBMCs from atherosclerotic patients. Moreover, an inflammatory challenge (TNF-*α*) also suppressed klotho expression in primary HUVECs. These results indicated a potential that klotho reduction may promote inflammation. To test this hypothesis, klotho was overexpressed in vascular endothelial cells. Interestingly, klotho overexpression significantly decreased IL-6 secretion. Following a challenge of TNF-*α*, the elevated IL-6 production was also markedly blunted in klotho-overexpressed cells. These data indicated that klotho has a potent role in inhibiting IL-6 production. Furthermore, we examined the regulatory effect of pravastatin on klotho expression in HUVECs. As expected, pravastatin dose-dependently and markedly upregulated klotho in HUVECs, which indicated that the inhibitory effect of pravastatin on IL-6 production could be resulting from its action on inducing klotho in vascular cells at least to some extent. These findings also suggested that the beneficial effects of pravastatin on cardiovascular system might be partially from its inhibition on IL-6 in circulation and atherosclerotic regions.

In summary, using primary HUVECs and mononuclear cells from patients and genetic approaches, we studied pravastatin effect on regulating klotho and IL-6 and the relationships between them. The results indicated that upregulation of klotho and subsequent suppression of IL-6 might be an important mechanism of pravastatin in protecting cardiovascular system. The downregulation of klotho in atherosclerotic patients could be involved in the high circulating IL-6 level in these subjects. Therapies by targeting vascular and inflammatory cell klotho might be promising for the treatment of atherosclerotic diseases.
